# GENDER DIFFERENCE IN RETIREMENT TIMING

**DOI:** 10.1093/geroni/igz038.1103

**Published:** 2019-11-08

**Authors:** Hansol Kim, Hyun Kang, David J Ekerdt

**Affiliations:** 1 Gerontology, University of Kansas, Lawrence, Kansas, United States; 2 University of Kansas, Lawrence, Kansas, United States

## Abstract

The aim of this research is to examine the retirement timing of older men and women in the United States and to find what factors impact such timings. This research used the 2014 Health and Retirement Study datasets. A total of 2,401 respondents were included in this research. All of the participants were over 60 years old, half were women, and the majority of participants were full-time workers (81.8%). The dependent variable was expected years until retirement which was measured as a continuous variable, asking when the respondent thinks he/she will stop work or retire. Controlling for age, race, marital status, education, health, full time, and a number of children, the results revealed that males expect to work 1.2 years longer than women. Yet women have reasons for working longer that are not found among men. Older age and poor health predict a sooner retirement for both men and women. Yet women differed from men in wanting longer work lives if they are African American, employed part-time, and have large families. Women are living longer than men, and the labor participation of women is increasing. Older women will have more challenge in preparing for retirement than men due to their greater need to extend work to secure income. Gender differences in expectation for retirement financial security and their effect on retirement timing. Deserves future research, to understand women’s decision making at this life stage.

